# Psittacine Beak and Feather Disease: Global Spread, International Trade, and Conservation Challenges

**DOI:** 10.3390/ani15202947

**Published:** 2025-10-10

**Authors:** Eun Gu Kang, Jang-Hee Han, Yong Ju Shim, Do Na Lee, Kang-Seuk Choi, Seong-Chan Yeon

**Affiliations:** 1 Department of Veterinary Clinical Sciences, College of Veterinary Medicine and Research Institute for Veterinary Science, Seoul National University, Seoul 08826, Republic of Korea; vet_kang@snu.ac.kr (E.G.K.); hjh5291996@snu.ac.kr (J.-H.H.); leedona@snu.ac.kr (D.N.L.); 2 Laboratory of Avian Diseases and Research Institute for Veterinary Science, College of Veterinary Medicine, Seoul National University, Seoul 08826, Republic of Korea

**Keywords:** psittacine beak and feather disease (PBFD), *Circovirus parrot*, transmission, diagnosis, clinical signs, parrot conservation, wildlife trade, conservation policy, biosecurity protocol, prophylaxis

## Abstract

**Simple Summary:**

Psittacine Beak and Feather Disease (PBFD) is a major, often fatal infectious disease in parrots caused by a highly stable circovirus, *Circovirus parrot*. First identified in Australia in the 1880s, it now poses a significant global threat with no definitive cure or commercially available vaccine to date. This creates a complex challenge, as parrots are popular pets in a vast global trade, while also representing a high conservation priority, with over a third of all species threatened. This review provides a comprehensive analysis of PBFD, synthesizing up-to-date information to support critical decisions in veterinary care, conservation, and aviculture. What distinguishes this work is its in-depth investigation of the international parrot trade as the primary driver of the disease’s global dissemination, fueled by both legal and illegal activities. The analysis thoroughly assesses the ecological and conservation consequences of sustained viral circulation in both captive and wild populations. It also integrates current vaccine research within a policy-relevant framework to propose future management strategies for these trade-related challenges. By merging veterinary science, aviculture, and conservation biology, this integrated approach delivers profound insights not previously consolidated in a single work, marking its significant contribution to the field.

**Abstract:**

Psittacine Beak and Feather Disease (PBFD) is a highly contagious viral condition caused by *Circovirus parrot*—commonly known as Beak and Feather Disease Virus (BFDV)—a small, single-stranded DNA virus of the family *Circoviridae*. The disease primarily affects parrots (order *Psittaciformes*) and is characterized by progressive feather dystrophy, beak deformities, immunosuppression, and high mortality rates, particularly in juvenile birds. Although PBFD was initially documented in *Australian psittacines*, the virus has now attained global distribution, facilitated predominantly by the international trade in live parrots, both legal and illegal. This review provides a comprehensive synthesis of current knowledge on the virology, clinical presentation, molecular epidemiology, and phylogeographic spread of BFDV. Particular attention is given to the role of parrot trade in shaping transmission dynamics and genetic diversification. The review further evaluates existing biosecurity policies, diagnostic challenges, and disease management strategies within both captive and wild avian contexts. Given PBFD’s dual status as a veterinary concern and a growing conservation threat, strengthening international surveillance, regulating wildlife trade, and integrating molecular diagnostics into routine screening are critical priorities. Effective containment of BFDV requires a multidisciplinary approach involving veterinarians, aviculturists, conservation biologists, and policymakers to safeguard the health and genetic viability of endangered psittacine species globally.

## 1. Introduction

Psittacine beak and feather disease (PBFD) is a highly contagious condition caused by beak and feather disease virus (BFDV), posing significant threats to global parrot populations [1]. Clinically characterized by dystrophic feathering and progressive beak deformities in psittacine species, PBFD was first observed in Australian wild red-rumped parrots (*Psephotus haematonotus*) in 1907 [2,3,4]. It was later formally recognized as a distinct disease entity affecting multiple psittacine taxa in the 1970s [3].

Initial hypotheses regarding its etiology speculated nutritional deficiencies, endocrine disorders, and bacterial or fungal infections [4]. However, in the early 1980s, a novel circovirus was identified as the causative agent, and was subsequently named BFDV, which belongs to the *Circoviridae* family [5,6]. Following the identification of the virus, PBFD disseminated globally via international trade in psittacine birds, either legal or illegal [7]. To date, it has been documented in both captive and free-ranging birds across over 40 countries, with evidence of high genetic variability, especially in wild populations [8].

Recent research has further expanded the recognized host range of BFDV beyond Psittaciformes. Viral DNA has been detected in at least eight additional avian orders, including *Coraciiformes* [9], *Strigiformes* [10], and *Passeriformes* [11], indicating broader ecological implications and interspecies transmission potential.

Due to the complexity of producing an effective vaccine or targeted antiviral therapy, BFDV continues to spread both among and within species, circulating in wild and captive populations [12]. This persistent transmission presents significant challenges for avian conservation, particularly in endangered or geographically isolated psittacine species, where virus introduction may accelerate population decline and undermine recovery programs [13]. Subclinical infections and long incubation periods also limit screening effectiveness. These factors highlight the importance of integrating pathogen surveillance into conservation strategies [14].

This review synthesizes the current understanding of BFDV, focusing on its molecular biology, transmission dynamics, diagnostic approaches, and challenges hindering vaccine development. By integrating recent findings, we emphasize the urgent need for enhanced surveillance, robust biosecurity protocols, and targeted conservation efforts to mitigate ongoing viral impact on global avian biodiversity.

## 2. Literature Search

### 2.1. Publised Date Range

This review prioritizes literature published within the last 20 years to ensure currency. However, foundational papers concerning the discovery, history, or established principles of PBFD were included regardless of their publication date. For data from major organizations and government bodies, such as the International Union for Conservation of Nature (IUCN), the International Committee on Taxonomy of Viruses (ICTV), and the Australian government, the most recent information available as of 1 July 2024, was referenced.

### 2.2. Search Parameters and Limitation

The literature search was conducted primarily on the Google Scholar and PubMed databases and was restricted to English-language articles to ensure objectivity and accessibility. In instances where peer-reviewed data were limited, such as for very recent case reports, credible news sources and web-based resources were referenced.

It is acknowledged that local-language resources may exist in regions with significant interest in parrots, such as Africa and South America, which were likely omitted due to language and access constraints. Furthermore, while a substantial body of empirical data on parrot care is shared within breeder communities, this information generally lacks scientific rigor and is not organized according to the scientific method. Consequently, such anecdotal data were deemed unsuitable for inclusion in this review.

## 3. Taxonomy and Nomenclature

### 3.1. Taxonomy

The causative agent of PBFD, designated by the International Committee on Taxonomy of Viruses (ICTV) as *Circovirus parrot*, is a member of the family *Circoviridae*. This family comprises small, autonomously replicating viruses characterized by a circular single-stranded DNA (ssDNA) genome [5]. Despite reports describing a high degree of genetic variation in *Circovirus parrot* among psittacine birds, such differences are not formally acknowledged by the ICTV as distinct genotypes [8,15]. Rather, they are generally considered to represent genetic polymorphisms within a single established genotype [16]. To date, no particular variant has been definitively associated with higher virulence or species-specific disease outcomes. Nevertheless, numerous avian viruses within the genus *Circovirus*, such as the zebra finch circovirus, have been newly identified in recent years, and the continued advancement of next-generation sequencing (NGS) and metagenomic approaches is expected to uncover many more in the future [17].

Other notable circoviruses include porcine circoviruses (PCV1 and PCV2), which are economically significant pathogens in swine production [18]. Due to its immunosuppressive effects and impact on avian biodiversity, BFDV continues to be a focal point of molecular virology and conservation research [19].

### 3.2. Nomenclature of PBFD and BFDV

#### PBFD (Disease Terminology)

The disease now referred to as PBFD was initially identified based on its characteristic clinical signs: symmetrical feather dystrophy, progressive beak deformities, and immune suppression in psittacine birds [4,20]. Early veterinary literature used varied terms such as “beak and feather disease,” “psittacine feather dystrophy,” and “parrot feather disease,” reflecting descriptive impressions rather than a unified pathological framework.

Due to overlapping signs with other dermatological or behavioral disorders (e.g., feather picking or beak trauma), some early cases were misclassified under informal terms like “beak rot” or “Galah syndrome” [20]. It was only through improved pathological and virological insight—including detection of intranuclear inclusion bodies and circoviral particles—that PBFD became consistently defined as a distinct clinical entity.

## 4. Virus Structure and Replication

### 4.1. Virus Structure

**Figure 1:**
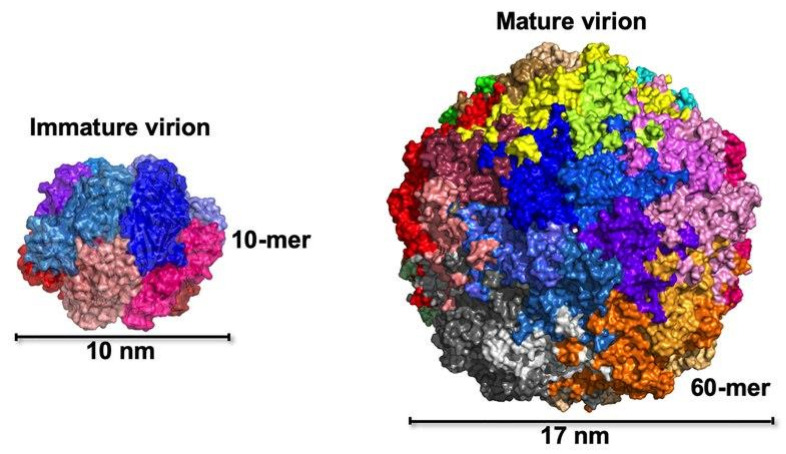
Modeling of 10 nm immature virions (**left**) and 17 nm mature virions (**right**). High-resolution X-ray crystallography revealed the structures of two BFDV capsid virions. The immature virion, about 10 nm in diameter and modeled at 1.9 Å resolution, consists of 10 capsid proteins arranged into two interlocking disks (each disk contains five proteins). In contrast, the mature virion, approximately 17 nm wide and resolved at 2.5 Å, is composed of 60 capsid proteins organized into 12 pentameric units with T = 1 icosahedral symmetry. Sarker et al., CC-BY 4.0 [21].

BFDV is a non-enveloped, circular ssDNA virus with a small icosahedral capsid measuring approximately 14–20 nm in diameter, exhibiting T = 1 symmetry as shown in Figure 1 [21]. Detailed ultrastructural studies using electron microscopy and crystallography have revealed multiple assembly states of the capsid protein (Cap) during infection. These include cytoplasmic aggregates (~0.1–0.5 µm), membrane-bound inclusions (~0.5–5.0 µm), intranuclear assemblies (~10–12 nm), and mature virions (~17 nm) [22,23]. Unlike most non-enveloped DNA viruses, BFDV forms dense cytoplasmic paracrystalline arrays. It does not accumulate solely in the nucleus. These patterns offer insight into intracellular trafficking and capsid maturation.

High-resolution crystallographic analyzes identified two major Cap protein assemblies: a ~10 nm decameric intermediate formed in the absence of DNA, and a ~17 nm mature capsid stabilized by interactions between the positively charged N-terminal arginine-rich motifs (ARMs) and the negatively charged viral genome [21]. Each Cap monomer adopts a canonical viral jelly-roll β-barrel fold, consisting of two β-sheets (BIDG and CHEF), which interlock to provide mechanical stability to the capsid.

### 4.2. Genome Structure and Gene Function

**Figure 2:**
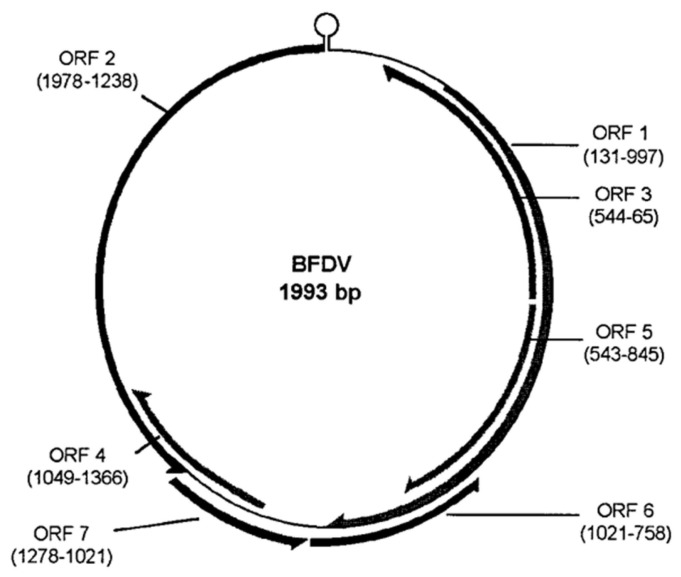
Schematic diagram of the BFDV genome Seven ORFs. Bassami et al., CC-BY 4.0 [19].

The BFDV genome is a circular single-stranded DNA (ssDNA) molecule approximately 2 kilobases in length as shown in Figure 2 [19,24]. It encodes at least three major open reading frames (ORFs): the replication-associated protein (Rep), the capsid protein (Cap), and a third ORF of currently undefined function [18,19]. As with other members of the *Circoviridae* family, BFDV lacks its own DNA polymerase and relies entirely on host polymerase activity for genome replication [25].

The Rep protein initiates and regulates viral replication via a rolling-circle replication (RCR) mechanism [26]. This involves recognition and cleavage at a specific stem-loop origin, strand displacement, and synthesis of the complementary strand using host DNA polymerases [27].

The Cap protein fulfills both structural and non-structural roles. Structurally, it forms the viral icosahedral capsid that encases the genome. Functionally, Cap binds ssDNA, interacts with host cell transport machinery (e.g., dynein/microtubule complexes), and contains nuclear localization signals (NLS) within its arginine-rich motifs (ARMs), facilitating nuclear import via the importin-α/β pathway [21]. Assembly of mature capsids occurs within the nucleus when Cap associates with replicated viral DNA.

Additionally, the circular ssDNA genome contributes to virion stability by neutralizing the positive charges within the capsid, promoting efficient assembly. Cap monomers initially form small cytoplasmic oligomers that can translocate to the nucleus. There, encapsidation of the viral genome induces the final assembly of infectious virions. This modular and flexible assembly mechanism underlies the persistence and adaptability of BFDV in diverse host environments.

### 4.3. Replication Cycle

The replication cycle of BFDV begins with the entry of viral particles into the host cell, followed by nuclear import of the viral genome and associated replication proteins. The capsid protein (Cap) plays a pivotal role in this process, acting as a shuttle for both the viral genome and Rep protein into the nucleus. This nuclear localization is mediated by arginine-rich motifs and overlapping nuclear localization signals (NLS) in the N-terminus of Cap [25].

Once inside the nucleus, the circular ssDNA genome is converted to a double-stranded DNA (dsDNA) intermediate, which serves as a template for rolling-circle replication (RCR). The Rep protein recognizes conserved origin motifs, initiates strand cleavage via endonuclease activity, and facilitates DNA unwinding and synthesis using host DNA polymerases [28]. Rep also exhibits ATPase and GTPase activities, which regulate DNA binding and processing during replication [29].

Replication primarily occurs in lymphoid tissues such as the bursa of Fabricius and gut-associated lymphoid tissue, followed by systemic dissemination to the liver, thymus, and bone marrow. Secondary replication in feather follicle keratinocytes supports high-level viral production and accounts for the feather abnormalities seen in PBFD [24].

## 5. Host Range, Virulence and Pathogenesis

### 5.1. Host Range

BFDV exhibits a broad and continually expanding host range. While its primary reservoir remains within psittacine species—including cockatoos, lorikeets, and parrots—it is now increasingly detected in non-psittacine avian orders [30]. This expanded host spectrum underscores the virus’s ecological plasticity and suggests potential for widespread interspecies transmission.

Recent phylogenetic and epidemiological studies have provided strong evidence of host-switching events among Australian parrot species and even between unrelated avian taxa, facilitated by overlapping habitats and shared environmental reservoirs [7]. Detection of BFDV in clinically unaffected individuals from non-psittacine orders further suggests possible subclinical or latent carriage in broader avian communities.

These findings challenge the previously held view of BFDV as a strictly psittacine pathogen and have significant implications for wildlife disease surveillance and avian conservation programs globally.

#### 5.1.1. Captive Psittacine Populations

Beak and feather disease virus (BFDV) is commonly reported in captive psittacine populations across diverse geographical regions. Its high transmissibility, capacity to induce immunosuppression, and association with reproductive failure present critical challenges in aviary settings. In Germany, one of the earliest epidemiological surveys reported a 39.2% prevalence of BFDV among asymptomatic captive parrots from multiple breeders [31].

Similarly, in Bangladesh, PCR screening of birds from breeding farms, markets, and household flocks revealed a 37% positivity rate, with the majority of infected individuals showing no clinical signs. These findings confirm widespread subclinical infections and underscore the risk posed by unregulated trade and mixed-species housing [32].

In Chile, the first molecular surveillance of captive exotic psittacines found a BFDV prevalence of 23.2% across 17 genera, further highlighting the virus’s broad host range and silent circulation within managed bird collections [33].

Together, these studies demonstrate that captive parrots, even when clinically normal, may function as persistent viral reservoirs and active sources of BFDV transmission in both domestic and international contexts.

#### 5.1.2. Wild *Psittacine* Populations

In wild psittacine populations, BFDV has been identified across multiple species—frequently in the absence of visible clinical signs. For instance, a longitudinal study of crimson rosellas (*Platycercus elegans*) revealed that over 40% of tested individuals were BFDV-positive, and most remained asymptomatic during the study period [34]. Notably, infected birds often cleared the virus from circulation within months, yet evidence of prior immunological responses persisted, suggesting transient or latent infections.

Broader field surveillance has confirmed high prevalence rates among wild parrots across Australia, with many positive individuals showing no signs of PBFD. One study found that up to 28.9% of wild crimson rosellas were coinfected with other pathogens like *C*. *psittaci*, despite appearing clinically healthy [34].

These findings challenge the assumption that PBFD is uniformly symptomatic in the wild and emphasize the role of subclinical carriers in maintaining viral persistence and environmental transmission across populations.

#### 5.1.3. Infections in Non-*Psittacine* Species

Although BFDV is classically associated with psittacine hosts, increasing evidence supports its presence in non-psittacine birds, raising important concerns regarding cross-order transmission and potential ecological spillover. A compelling example involves a spillover event into rainbow bee-eaters (*Merops ornatus*), a species from the order *Coraciiformes*, in which a self-limiting BFDV infection was confirmed using PCR and genomic analysis [9].

Similarly, forensic genomic analysis confirmed BFDV DNA in a powerful owl (*Ninox strenua*), likely acquired through predation on infected parrots or environmental contamination. This finding represents an unusual yet significant host jump into *Strigiformes*, with potential implications for raptor health [10].

Additionally, a recent report documented BFDV infection in the endangered red goshawk (*Erythrotriorchis radiatus*), marking the first detection of the virus in a non-psittacine raptor listed as endangered. This discovery suggests a broader host susceptibility than previously understood and highlights the potential for interspecies transmission via predation or environmental exposure [35].

Together, these findings indicate that BFDV’s ecological reach may be underestimated, warranting expanded surveillance beyond psittacines to better understand viral ecology, potential reservoir species, and risks to avian biodiversity.

### 5.2. Pathogenesis and Virulence

Beak and feather disease virus (BFDV) exhibits pronounced tropism for keratinized tissues, particularly developing feather follicles and beak epithelium. Following initial replication in the bursa of Fabricius, systemic viremia leads to dissemination to lymphoid organs such as the thymus, spleen, bone marrow, and liver, where BFDV induces cytopathic effects and profound immunosuppression [36].

Histopathological evidence confirms extensive lymphoid apoptosis and bone marrow suppression, particularly in juvenile birds. Observations include leukopenia, anemia, and severe lymphoid depletion, often attributed to caspase-dependent apoptosis within immune tissues [37]. Experimental infections also support these findings, showing increased apoptotic activity in the bursa and bone marrow with viral antigen localized within apoptotic cells. These pathologies are especially severe in neonates and juveniles, consistent with age-dependent immune system immaturity and increased susceptibility to immunosuppressive infections [37]. Similar apoptotic patterns are observed in related circoviruses, such as porcine circovirus 2 (PCV2), which triggers lymphoid depletion through caspase-3 and -8 activation [38].

Beyond immunosuppression, BFDV demonstrates remarkable environmental resilience. The virus is stable in dried feather dust and contaminated fomites, facilitating long-term horizontal transmission via direct contact or environmental exposure. Moreover, vertical transmission has been confirmed through PCR detection of BFDV DNA in embryonated eggs, complicating management in both captive and wild populations [36].

Critically, many asymptomatic birds function as persistent carriers, sustaining viral circulation without overt clinical signs. This latent infection potential, coupled with environmental durability, underscores the virus’s capacity for silent spread and long-term ecological persistence.

## 6. Epidemiology and Transmission

### 6.1. Epidemiology

**Figure 3:**
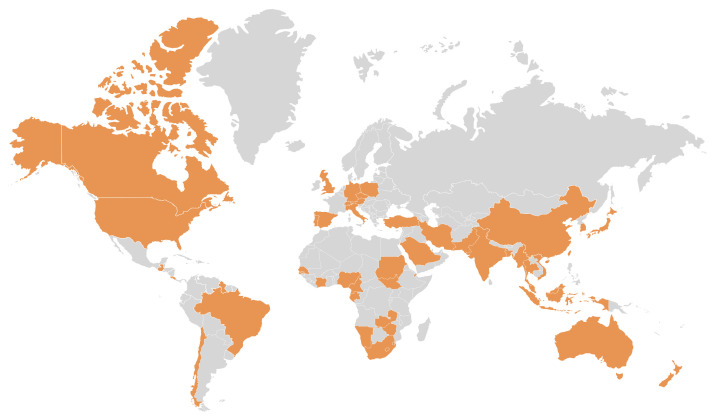
Global distribution of PBFD reports (orange color) since its first description in Australia in the 1980s [39,40,41,42,43,44,45].

As global surveillance of BFDV has intensified, infection cases are increasingly being identified in both free-living and captive parrots across multiple continents as shown in Figure 3. In some wild populations, BFDV prevalence has been reported at rates as high as 70%, and many infected individuals remain asymptomatic. These findings underscore the role of subclinical carriers in sustaining viral circulation and complicating epidemiological surveillance [39].

The primary driver of BFDV’s transcontinental spread has been the international trade of psittacines, both legal and illegal. Since the 1970s, millions of parrots have been moved globally, often without adequate health screening. This has facilitated the silent introduction of BFDV into new regions, where it has become established in both native and invasive avian populations [40].

Between 1975 and the early 2000s, over 19 million parrots were internationally traded, with limited biosecurity controls. Phylogeographic studies have revealed direct links between trade intensity and BFDV strain dissemination, including the movement of specific viral lineages across continents [41]. Island ecosystems, such as those in Seychelles and Mauritius, have proven especially vulnerable, highlighting the urgent need for globally harmonized regulations on parrot trade and improved disease surveillance systems.

Australia is widely accepted as the evolutionary origin of BFDV. Native Australian parrot species harbor diverse viral genotypes that are genetically distinct yet phylogenetically clustered, supporting a long co-evolutionary history [42]. Despite modern prohibitions on parrot exports, historical trade facilitated BFDV dissemination to nearby regions. For example, molecular analyzes of viral genomes from *Psittacula* and *Cyanoramphus* species in New Zealand and New Caledonia have revealed close genetic relationships to Australian BFDV strains, implicating unregulated wildlife trade and species translocation in the virus’s regional spread [44]. In New Caledonia, novel lineages such as the BFDV-P strain have emerged, though they still retain genomic motifs indicative of Australian *Loriinae* origins. These findings illustrate how legacy trade routes and invasive species introductions continue to shape BFDV’s global phylogeography. Without sustained biosecurity measures, the virus is likely to remain a persistent threat to conservation, captive breeding, and international avian commerce.

In Asia, BFDV infections are increasingly prevalent in countries such as Bangladesh, Pakistan, and Vietnam. The region is recognized as a hotspot for viral recombination and cross-species transmission, particularly involving the rose-ringed parakeet (*Psittacula krameri*), a widely traded and invasive species. Molecular studies show that *P. krameri* functions as a reservoir host, facilitating the spread of BFDV into native ecosystems. Notably, spillover has been observed in Vietnam, where *P. finschii* populations—already threatened by habitat loss—face additional pressure from BFDV introduced via the pet trade [40,44].

Across Africa, BFDV has been detected in both wild and captive psittacines. In South Africa, the virus poses a notable risk to the endangered Cape parrot (*Poicephalus robustus*) [46]. Phylogenetic analysis of viral isolates from West Africa, including Senegal and Gambia, indicates multiple introduction events, with strains genetically linked to Southeast Asian and Australasian BFDV lineages—strongly implicating illegal trade and unregulated imports [40,42].

Europe has long served as a hub in the international parrot trade, and although modern import regulations have restricted the influx of wild-caught birds, BFDV remains endemic in captive aviaries. Established feral populations of *P. krameri* in countries such as the United Kingdom harbor BFDV strains genetically similar to those circulating in Asia, reflecting historical trade origins [39]. These captive and feral populations act as viral reservoirs, posing persistent spillover risks to wild European avifauna.

In North America and parts of South America, stricter regulations have successfully limited direct introductions of infected wild parrots. However, BFDV persists within captive collections, where occasional lapses in biosecurity contribute to localized outbreaks. In Brazil, escaped or released captive parrots have introduced BFDV into wild settings, with genetic evidence tracing these strains back to European and Australasian origins [47].

Isolated island ecosystems are particularly vulnerable to viral invasions. In Mauritius, BFDV has been documented in both the endemic Mauritius parakeet (*Psittacula eques*) and invasive *P. krameri* populations, with introductions strongly tied to the international bird trade [40]. Similarly, in New Caledonia, at least two independent viral introductions have been identified, including a novel BFDV lineage, highlighting the risks posed by invasive species within fragile insular ecosystems [43].

Collectively, the widespread detection of BFDV in internationally traded parrots highlights the virus’s global epidemiological significance. Phylogenetic analyses consistently reveal limited geographic clustering among viral strains, indicating frequent long-distance dispersal mediated by both legal and illegal parrot trade activities [40]. The resulting global homogenization of BFDV genotypes, characterized by lineages originating from Asia, Africa, Oceania, and Europe appearing in geographically distant regions, underscores the profound influence of international avian trade networks on the virus’s global evolutionary dynamics.

Addressing the ongoing spread and ecological impacts of BFDV necessitates a coordinated international response involving stricter enforcement of CITES regulations to disrupt illegal wildlife trade pathways, standardized and enhanced quarantine and diagnostic protocols at import and export facilities, and comprehensive biosecurity improvements within captive breeding operations. These coordinated strategies are crucial for early detection, containment, and prevention of further viral dissemination across international borders [44]. Without such robust international collaboration and standardized biosecurity measures, BFDV will continue to compromise conservation breeding efforts, exacerbate viral transmission into wild bird populations, and intensify biodiversity declines, especially within ecologically vulnerable regions such as islands and biodiversity hotspots [42].

### 6.2. Transmission

#### 6.2.1. Vertical Transmission

Evidence increasingly supports the capacity of beak and feather disease virus (BFDV) to be transmitted vertically from infected parent birds to their offspring. Detection of BFDV DNA in both embryonated and non-embryonated eggs of psittacines indicates that transovarial transmission—direct infection of the developing embryo—is biologically plausible. For instance, in budgerigars (*Melopsittacus undulatus*), viral DNA was detected in 35.3% of non-embryonated and 20% of embryonated eggs, suggesting that in ovo infection can occur under natural or captive conditions [48].

Although these findings suggest genuine vertical transmission potential, distinguishing true prenatal infection from very early post-hatch exposure—such as via crop secretions, feather dust, or contaminated nesting material—remains methodologically challenging. Field evidence further complicates the interpretation: some nest monitoring studies report lower-than-expected infection rates among hatchlings of infected parents, indicating that not all parental infections result in vertical transfer.

Notably, research from Australia’s Orange-bellied Parrot (OBP; *Neophema chrysogaster*) recovery program has highlighted the risk of early-life BFDV acquisition and its devastating consequences for endangered populations. Infected OBP fledglings exhibited genetically distinct viral strains, and full-genome sequencing revealed multiple independent introductions of BFDV into the captive and wild OBP populations. These incursions are believed to have occurred in the absence of an endemic viral reservoir, thereby exposing immunologically naïve individuals to newly introduced strains. This pattern—loss of endogenous viral circulation followed by repeated external spillover—may simulate vertical or perinatal transmission dynamics in closed or recovering populations [12,13].

In response, OBP conservation protocols now include artificial incubation and hand-rearing of eggs laid by known-exposed parents. These precautionary methods aim to reduce exposure during the critical hatching period and illustrate the importance of managing both vertical and early horizontal transmission pathways in high-risk or endangered avian populations.

#### 6.2.2. Horizontal Transmission

Horizontal transmission is the primary mechanism by which beak and feather disease virus (BFDV) spreads among psittacines. Infected birds shed large quantities of viral particles through feather dander, feces, crop secretions, and sloughed epithelium. Transmission typically occurs via inhalation or ingestion of contaminated material and is significantly accelerated by close social behaviors such as mutual preening, communal feeding, and high-density housing in aviaries [36].

BFDV demonstrates remarkable environmental stability, with viable viral DNA persisting on surfaces including perches, nesting materials, and cage substrates for extended periods. A field-based environmental study detected BFDV DNA on nest box surfaces up to 3.7 months after initial contamination, confirming indirect fomite transmission as a critical and underappreciated route of infection [49]. Environmental viral load was closely associated with the infection status of adult birds occupying the nest box, especially during the breeding season.

The virus’s immunosuppressive effects further compound its transmissibility. Infected individuals are more susceptible to secondary pathogens, such as mites or fungal organisms, which may increase viral shedding and obscure clinical diagnosis. For instance, coinfection with *Knemidocoptes pilae* in a sulphur-crested cockatoo was associated with elevated BFDV antigen concentrations in gut contents and feces, raising concerns about the role of ectoparasites as mechanical vectors [50].

Given the virus’s environmental persistence and multiple transmission routes, comprehensive biosecurity protocols are essential. These include disinfection of aviary surfaces, equipment, and clothing with agents such as peroxygen-based disinfectants (e.g., Virkon S [36]), especially after confirmed PBFD cases. In both captive and conservation settings, strict hygiene practices are paramount to prevent horizontal spread and limit long-term establishment of the virus in enclosed populations.

### 6.3. Role of Asymptomatic Carriers in Viral Transmission

The widespread detection of BFDV in asymptomatic individuals—particularly in wild psittacine and non-psittacine species—suggests that subclinical infections play a central role in viral persistence and epidemiology. In crimson rosellas (*Platycercus elegans*), BFDV was found in over 90% of sampled birds when multiple tissues were examined, despite a lack of outward signs. Notably, the virus persisted at low levels in adult tissues, even among birds that had previously cleared viremia, indicating chronic tissue-level carriage [51].

Similarly, population-level studies have documented high prevalence of BFDV among apparently healthy birds. For example, up to 56.2% of psittacine and 20.0% of non-psittacine wild birds tested positive for BFDV in one Australian survey, despite an absence of clinical signs [30]. The silent carriage of BFDV complicates detection and control efforts, as conventional blood-based screening may miss tissue-localized infections. Further, the ecological role of asymptomatic carriers—especially non-psittacine hosts—remains unclear: whether they serve as true reservoirs or incidental dead-end hosts requires clarification through experimental and ecological studies. Understanding the immunological, virological, and behavioral dynamics of these subclinical infections is essential for evaluating BFDV’s potential for long-term persistence, cross-species transmission, and its implications for avian conservation programs.

## 7. Clinical Signs and Histopathology

### 7.1. Clinical Manifestations in Psittacines

PBFD manifests in two principal clinical forms—acute and chronic—each exhibiting distinct age-related patterns and pathophysiological outcomes as shown in Table 1.

Acute PBFD typically affects nestlings and recently fledged juvenile parrots. The disease course is rapid, with affected birds often exhibiting lethargy, anorexia, green diarrhea, and generalized weakness, frequently culminating in death before the onset of typical feather lesions. In African Grey parrots (*Psittacus erithacus*), acute cases have been reported where birds died suddenly without exhibiting visible feather or beak abnormalities, underscoring the diagnostic challenge in early-stage infections [52]. Necropsy findings often reveal hepatic necrosis, severe lymphoid depletion, pancytopenia, and non-regenerative anemia, consistent with profound bone marrow suppression and systemic immunosuppression [37].

Chronic PBFD is more commonly observed in older juveniles and adult parrots (typically between 6 months and 5 years of age). Clinical progression is slower and characterized by progressive symmetrical feather loss, starting with powder-down feathers and advancing to contour, tail, and flight feathers. Dystrophic feather growth is common, including retained sheaths, hemorrhagic shafts, feather necrosis, and malformed vanes. Progressive beak deformities, particularly elongation, softening, or necrosis of the upper beak, may also emerge. Chronic cases frequently present with self-trauma and secondary dermatitis, due to persistent follicular inflammation and pruritus.

Viral load has been shown to correlate inversely with clinical condition. In wild Cape parrots, higher BFDV DNA levels were associated with poorer physical condition and more overt clinical signs, affirming the link between viremia and disease severity [46]. Together, these findings underscore the spectrum of clinical presentations in PBFD, shaped by host species, age, immune status, and viral load. Early identification and differentiation of acute versus chronic presentations are critical for effective disease management and biosecurity response in both wild and captive psittacine populations.

Beak deformities are a prominent feature in chronic PBFD, particularly severe in cockatoos, presenting as elongated, fractured, or sloughed beaks (*rhamphotheca*). Additionally, hyperkeratosis of the skin on feet and legs, abnormal pigmentation, and chronic ulcers at pressure points (e.g., wing tips, elbows) can develop. Systemic signs such as weight loss, depression, and increased susceptibility to secondary infections (bacterial, fungal, and viral) frequently accompany chronic PBFD, ultimately leading to mortality due to secondary complications or cumulative metabolic stress.

Clinical severity varies with host factors (species, age), viral strain virulence, and exposure route. Old World parrots (e.g., cockatoos, African grey parrots, lovebirds) generally suffer more severe disease compared to New World parrots (macaws, Amazons, conures), which may harbor subclinical infections or exhibit milder clinical signs. Notably, cockatoos display pronounced feather loss and severe beak deformities, whereas African grey parrots often exhibit less severe feather lesions with unique features such as abnormal red feather pigmentation. Lovebirds (*Agapornis* spp.) typically present localized feather loss, with studies suggesting malnutrition may exacerbate clinical disease severity.

Although cockatiels (*Nymphicus hollandicus*) were historically considered resistant to PBFD, recent genomic and serological analyzes have confirmed infection with genetically distinct BFDV strains, indicating species-specific viral adaptation and the potential for subclinical or atypical presentations in this species [15].

Hematologically, acute PBFD is associated with marked leukopenia, whereas chronic forms frequently exhibit decreased serum proteins [52,53]. Histopathological findings consistently reveal epithelial and follicular necrosis, distal feather pulp degeneration, hemorrhage within feather calamus vessels, epidermal hyperplasia, and hyperkeratosis [6,54]. Characteristic intracytoplasmic and intranuclear basophilic viral inclusion bodies occur within feather follicle epithelium and associated macrophages [1]. Similar epithelial degeneration and necrosis extend to beak tissues, sometimes progressing to extensive sloughing [6]. Internal lesions include hepatic congestion, multifocal hepatic necrosis with inclusion bodies, thymic atrophy, and necrosis of the bursa of Fabricius, underlying severe immunosuppression [55]. Immunohistochemistry and in situ hybridization effectively demonstrate BFDV presence and tissue distribution, confirming the systemic nature of infection [56,57].

### 7.2. Experimental Infections and Disease in Non-Psittacines

Experimental infections of beak and feather disease virus (BFDV) in non-psittacine avian species have produced variable outcomes, suggesting that host susceptibility may be species-specific and modulated by ecological or immunological context. For instance, inoculation of specific-pathogen-free chickens (*Gallus gallus domesticus*) via oral, subcutaneous, and cloacal routes failed to induce observable clinical or pathological signs, indicating either innate resistance or restricted viral replication in galliform hosts [36].

However, accumulating field evidence challenges the presumption that non-psittacine birds are universally refractory to BFDV-induced disease. A notable example includes a self-limiting natural infection in rainbow bee-eaters (*Merops ornatus*), a species within *Coraciiformes*, which displayed mild feather abnormalities. Molecular analysis confirmed the presence of BFDV, suggesting a rare but definitive host-switch event outside the order Psittaciformes [9].

Similarly, forensic molecular diagnostics identified BFDV DNA in the tissues of a powerful owl (*Ninox strenua*), a non-psittacine predator species. The owl presented with hepatic and follicular lesions suggestive of PBFD-like pathology, and sequence data implicated *Trichoglossus* spp. (lorikeets) as the likely source of viral transmission via predation [10]. Further reinforcing these findings, a boobook owl (*Ninox boobook*) was also found to harbor a full-length BFDV genome, representing an additional host record in a non-psittacine raptor [58].

Beyond avian predators, evidence of BFDV presence in Gouldian finches (*Erythrura gouldiae*)—passerine birds unrelated to parrots—has been supported by histopathology and PCR detection. These individuals exhibited clinical signs such as feather dystrophy, suggesting susceptibility and possible replication-competent infection, though additional controlled studies are needed to distinguish between transient carriage and pathogenicity.

The identification of novel circoviruses in non-psittacine species with PBFD-like lesions further complicates diagnostic clarity. For example, raven circovirus (RaCV) in Australian ravens (*Corvus coronoides*) and pigeon circovirus (PiCV) in Columbiformes display overlapping pathology with BFDV, including feather follicle degeneration, immunosuppression, and secondary infections [36]. These observations suggest that the *Circoviridae* family possesses a broader host plasticity and shared virulence traits than previously recognized. Additionally, metagenomic screening has detected BFDV DNA in unrelated species such as Canada geese (*Branta canadensis*) and common starlings (*Sturnus vulgaris*), though the significance of these detections remains uncertain and may reflect environmental contamination or low-grade spillover [9].

In conclusion, while classic PBFD remains primarily a disease of psittacine birds, the growing body of evidence pointing to spillover infections, cross-order transmission, and non-psittacine host involvement calls for expanded pathogen surveillance and targeted ecological risk assessments. Understanding the extent of BFDV’s host range is essential to inform biosecurity measures and predict the impact of viral emergence in non-traditional avian hosts.

## 8. Diagnosis

Accurate diagnosis of PBFD remains challenging, typically necessitating both clinical evaluation and laboratory confirmation. Although clinical signs and histopathology strongly suggest PBFD, these alone are insufficient, as various conditions—including feather picking, malnutrition, and polyomavirus infections—can mimic similar clinical presentations. Additionally, clinical evaluation may miss asymptomatic carriers, emphasizing the critical role of laboratory diagnostics.

### 8.1. Molecular Diagnostics

Molecular techniques targeting BFDV DNA currently represent the gold standard for definitive PBFD diagnosis, offering superior sensitivity and specificity over conventional histopathology or serological methods. Among these, polymerase chain reaction (PCR) has emerged as the most widely adopted tool for both clinical and epidemiological applications.

Initially, in situ hybridization was developed as a sensitive method to detect BFDV DNA directly in tissue samples, especially when viral inclusion bodies were not readily visible. Despite its diagnostic value, its limited scalability, high labor demands, and cost have prevented widespread adoption for routine screening [56].

By contrast, PCR-based assays offer a highly sensitive and specific means of detecting BFDV across various sample types—including whole blood, feather pulp, cloacal swabs, and tissue biopsies. PCR has been shown to outperform hemagglutination (HA) and hemagglutination inhibition (HI) assays in both subclinical and clinical contexts [14,59].

The choice of sample type significantly impacts diagnostic sensitivity and specificity:

- Blood samples, especially whole blood or buffy coat, are ideal for detecting cell-associated viremia with high accuracy and low environmental contamination risk. These are recommended for definitive diagnostics during active infection [60,61].
- Feather pulp samples are widely used due to ease of collection and relatively high viral loads, particularly in actively growing feathers. However, their reliability decreases during non-molting periods or in samples exposed to environmental contamination [62].
- Cloacal and buccal swabs provide practical non-invasive options, though sensitivity varies based on viral shedding dynamics and sample quality [63].
- Fecal samples are primarily suited for flock-level surveillance; however, they are prone to PCR inhibition and environmental degradation of viral nucleic acids [36].
- Tissue biopsies (either ante-mortem or post-mortem) offer conclusive diagnosis when molecular results are correlated with characteristic histopathological findings. In advanced disease, the virus is typically distributed across multiple organs including liver, spleen, and feather follicles [36].

A comparative sensitivity study in budgerigars revealed that PCR detection rates were highest from feather samples (especially blood feathers), followed by cloacal swabs, with blood samples being least frequently positive. These findings reflect the transient nature of viremia and highlight the necessity for strategic sampling based on clinical phase [62]. Moreover, PCR assay performance varies across laboratories, underscoring the need for standardization. In an inter-laboratory comparison, diagnostic accuracy ranged from 71% to 100%, with one commercial lab showing a 20% specificity, suggesting high false-positive rates [64]. High-resolution melt (HRM) PCR assays targeting the cap and rep genes of BFDV have further enhanced diagnostic resolution by allowing genotypic discrimination among BFDV strains. HRM-cap PCR showed high specificity (99.9%) and excellent agreement with conventional PCR, making it a valuable tool for both diagnosis and molecular epidemiology [63]. To improve diagnostic yield, a dual-sample approach—combining blood and feather pulp—is increasingly recommended in clinical practice. This strategy enables detection during both viremic and follicular replication phases, capturing a broader infection window [12,14].

Finally, all PCR-based methods require rigorous nucleic acid extraction protocols and appropriate internal controls to prevent false-negative results from inhibitors commonly present in fecal or blood samples. When initial results are discordant with clinical suspicion, repeat testing with alternative sample types or methods is strongly advised.

### 8.2. Advanced Molecular Techniques

To overcome the known limitations of conventional PCR—such as sequence mismatch sensitivity, endpoint analysis constraints, and inability to quantify viral burden—several advanced molecular techniques have been developed and validated for the detection, characterization, and genotyping of BFDV. These techniques have enhanced diagnostic resolution, particularly in epidemiological surveillance, vaccine trials, and molecular pathogenesis studies.

#### 8.2.1. Rolling Circle Amplification (RCA)

Rolling circle amplification (RCA) is an isothermal, polymerase-driven reaction that amplifies circular DNA genomes such as those of circoviruses. RCA has proven particularly valuable for detecting BFDV DNA when sequence variation may hinder standard PCR primer binding [65]. When combined with conventional PCR (RCA-PCR), this approach enables both broad detection and downstream strain typing. However, RCA remains highly sensitive to sample quality and is susceptible to inhibition by degraded or contaminated nucleic acids. Moreover, definitive diagnosis typically requires additional sequencing or probe-based confirmation to distinguish BFDV from other circular DNA viruses or background amplification.

#### 8.2.2. Real-Time Quantitative PCR (RT-qPCR)

Quantitative PCR (qPCR) assays have become essential tools in both diagnostic and research contexts. Using intercalating dyes such as SYBR Green or sequence-specific fluorescent probes, qPCR enables the simultaneous detection and quantification of viral load, offering insight into infection kinetics, disease progression, and response to vaccination [66]. qPCR is particularly useful for tracking viremia in longitudinal studies or experimental infections.

Despite its advantages, qPCR sensitivity is highly dependent on DNA integrity and purification quality. Fecal and feather samples often contain PCR inhibitors, such as bile salts or keratin residues, that can reduce amplification efficiency or yield false-negative results [67]. Thus, proper extraction protocols and inclusion of internal amplification controls are essential.

#### 8.2.3. High-Resolution Melting Analysis (HRM)

High-resolution melting (HRM) is a post-PCR technique that identifies genetic variants by analyzing DNA melting profiles in the presence of fluorescent dyes. In BFDV research, HRM has enabled rapid, cost-effective genotyping without sequencing, providing an accessible tool for molecular epidemiology and intra-host diversity studies [63].

Targeting conserved genomic regions such as rep and cap, HRM has demonstrated high specificity (up to 99.9%) and excellent concordance with phylogenetic results. Nevertheless, its effectiveness is contingent on high-quality template DNA and specialized equipment, limiting its deployment in low-resource or field settings [63].

In summary, while PCR remains the primary tool for BFDV detection, advanced techniques such as RCA, qPCR, and HRM offer substantial advantages in sensitivity, quantification, and molecular typing. Their integration into surveillance and research workflows enhances the accuracy and depth of virological investigations, particularly for endangered or high-value avian populations. In addition to these methods, emerging technologies are poised to further revolutionize BFDV diagnostics. NGS, while still primarily a research tool, offers unparalleled depth for discovering novel viral genotypes, tracking micro-evolution during outbreaks, and conducting metagenomic surveillance in new or unexpected host species [17]. Furthermore, CRISPR-based diagnostic systems (such as SHERLOCK or DETECTR) are showing immense promise for the future [68,69,70]. These platforms could lead to the development of highly sensitive, specific, and rapid point-of-care tests that can be deployed directly in the field, overcoming the limitations of laboratory-based diagnostics. While specific applications for BFDV are still in early stages, these advanced molecular tools represent a critical frontier in the effort to monitor and control this global pathogen.

### 8.3. Integrated Diagnostic Strategies

Given the inherent limitations of individual diagnostic modalities, an integrated testing approach is strongly recommended to enhance diagnostic accuracy for PBFD. Isolated use of any single method may yield false-negative or ambiguous results, especially in asymptomatic carriers or birds with atypical presentations. As such, combining molecular, serological, and histopathological tools—alongside diverse sample types—substantially improves the reliability of detection and interpretation.

In cases where initial PCR results are negative but clinical suspicion remains high, clinicians are advised to:

- Repeat testing with a different sample type (e.g., from blood to feather pulp);
- Employ alternative PCR primers or confirmatory tests;
- Implement quantitative PCR (qPCR) to monitor viral dynamics over time.

For flock-level surveillance, a representative sampling strategy involving multiple birds and sample types (e.g., blood, feathers, environmental swabs) is crucial to account for variable viral shedding patterns and reduce the risk of underestimating infection prevalence. Table 2 provides a consolidated comparison of current diagnostic modalities, highlighting their respective roles, advantages, and constraints.

## 9. Management: Therapeutics, Prevention, and Vaccination

### 9.1. Therapeutics

Despite intensive research, no curative or targeted antiviral therapy currently exists for PBFD. Management strategies remain largely supportive, aimed at improving host condition, limiting secondary infections, and maintaining rigorous husbandry and biosecurity practices [72]. Experimental immunomodulatory approaches such as interferon therapy have demonstrated partial efficacy in reducing viral loads in selected cases; however, results remain inconsistent and unvalidated in controlled trials.

As a result, therapeutic emphasis continues to rest on supportive care, early detection, and robust preventive strategies, particularly in conservation or high-value aviary settings [73,74].

#### 9.1.1. Core Supportive Measures

Optimized Nutrition: A nutritionally balanced diet enriched with vitamin A, B-complex, and essential trace minerals supports immune function and feather regeneration. Birds with PBFD often benefit from dietary adjustments that reduce metabolic stress and promote epithelial integrity [75].

Hydration and Electrolyte Support: Dehydration, especially in cases with diarrhea or inappetence, is common. Fluid therapy via oral or subcutaneous routes restores volume and electrolyte balance; protocols are individualized based on clinical severity [76].

Secondary Infection Control: PBFD-associated immunosuppression significantly increases susceptibility to opportunistic bacterial, fungal, and parasitic pathogens. Timely antimicrobial therapy—based on cytology, culture, or empirical risk—is critical for prolonging survival [77].

Environmental Support and Hygiene: Due to compromised thermoregulation from feather loss, maintaining a stable, warm environment is vital. Rigorous hygiene reduces pathogen exposure and viral burden, especially in multi-bird or breeding facilities [74,78].

Symptomatic Relief: Birds with beak deformities often require regular trimming or corrective shaping to preserve feeding ability. Analgesics and anti-inflammatory agents may be administered for pain management, especially in cases involving cysts, severe feather follicle inflammation, or self-trauma [72].

While these interventions do not eliminate BFDV, they can significantly improve life quality and longevity. Many infected birds may survive for extended periods—particularly in stable, stress-reduced environments—succumbing only when secondary diseases overwhelm residual immunity.

#### 9.1.2. Experimental and Supportive Therapeutic Strategies

While supportive care remains the cornerstone of PBFD management, several experimental antiviral, immunomodulatory, and hematopoietic interventions have been explored. Although most remain in preclinical or anecdotal stages, these strategies offer conceptual frameworks for future therapeutic innovation.

### 9.2. Experimental Antiviral Agents

Several compounds have demonstrated in vitro efficacy or theoretical mechanisms against BFDV:

- Sodium orthovanadate (Na_3_VO_4_): Inhibits the ATPase activity of the BFDV replication-associated protein (Rep), effectively suppressing viral replication in vitro [74,79].
- L-742001: A Rep endonuclease inhibitor conceptually derived from circovirus models. Although not tested in *psittacines*, it may interfere with critical replication domains [28].
- Ribavirin: A broad-spectrum nucleoside analog that impairs RNA and DNA virus replication. In vitro studies in related viruses (e.g., porcine nidovirus) show suppression, but avian safety and efficacy remain unvalidated [80].

### 9.3. Immunomodulatory Treatments

Instead of directly targeting the virus, several therapies seek to boost host immunity:

- Avian interferon-gamma (IFN-γ): A key cytokine in antiviral defense. Anecdotal improvements have been reported in African grey parrots, but some studies caution that IFNs may paradoxically exacerbate circoviral replication [72].
- β-(1,3/1,6)-D-glucan: A fungal polysaccharide known to enhance innate immunity. Its use in cockatoos and horned parakeets (*Eunymphicus cornutus*) correlated with reduced viral DNA loads and improved feather condition, though data remain uncontrolled [81].
- Experimental hypothermia: Hypothesized to suppress systemic inflammation but remains speculative and untested in birds.
- Natural recovery: Documented in certain species, notably lorikeets and Eclectus parrots, some birds are able to clear infection or suppress clinical signs—likely via robust immune responses or antibody production [14].

### 9.4. Hematopoietic Support

PBFD is associated with marked leukopenia, and stimulating bone marrow activity may offer therapeutic benefit:

- Filgrastim (G-CSF): A human granulocyte colony-stimulating factor used in mammals for neutropenia. Its use in PBFD remains anecdotal, with some reports of transient leukocyte count improvement [82].
- Combined off-label regimens: A non-peer-reviewed case series reported a 70% BFDV PCR clearance rate using avian IFN-γ (10^6^ IU IM daily for 90 days) and nebulized F10^®^ disinfectant (1:125 dilution, 15 min daily). While intriguing, these results lack formal clinical validation, and the safety of nebulized disinfectant exposure in birds remains unproven [83].

### 9.5. Cross-Species Antivirals (Insights from PCV2 Models)

Given the structural similarity between BFDV and porcine circovirus type 2 (PCV2), compounds active against PCV2 are being considered for psittacine application. Epigallocatechin gallate (EGCG), matrine, and scutellarin, derived from Traditional Chinese Medicine (TCM), have demonstrated antiviral and immunomodulatory activity against PCV2 in vitro [24]. However, their bioavailability, dosing, and safety in parrots remain unstudied.

### 9.6. Prevention and Biosecurity

Preventing the transmission of BFDV requires an integrated approach encompassing biosecurity measures, hygiene protocols, routine screening, and eventually, vaccine development. BFDV’s resistance to environmental extremes and disinfectants complicates control efforts; however, several effective strategies exist:

- Disinfection: Beak and feather disease virus (BFDV) is notably resilient, persisting in the environment for extended periods. However, peroxygen-based disinfectants such as Virkon^®^ S (1%) have demonstrated efficacy against circoviruses and are recommended in biosecurity protocols, particularly by Australian wildlife agencies [84]. Quaternary ammonium compounds (e.g., Virex^®^) have also been successfully used in field settings, including nest site sanitation. Regular disinfection of all surfaces—including cages, perches, bowls, and equipment—is critical in multi-bird environments.
- Quarantine and Testing: Quarantine is foundational for PBFD prevention. New birds should be isolated for 30–45 days and tested for BFDV using multiple diagnostics: PCR on blood and feathers, and where possible, hemagglutination (HA) and hemagglutination inhibition (HI) assays, which enhance detection sensitivity, especially in early or subclinical infections [14,20]. Physical separation and the use of dedicated clothing/equipment within quarantine zones are essential.
- Routine Screening: Because PBFD can be asymptomatic, regular flock surveillance (every 6–12 months) is vital to detect latent infections before transmission occurs. Combining feather and blood PCR increases diagnostic reliability [59].
- Strict Access Control: Facilities should enforce biosecurity protocols, including footbaths, hand hygiene, and controlled visitor access. Equipment and gloves must be changed between enclosures to avoid cross-contamination. Segregating susceptible species like cockatoos and African grey parrots from potentially infected birds is strongly advised [84].
- Environmental Hygiene: PBFD virus is shed in feather dust, feces, and crop secretions [20]. To reduce airborne spread, HEPA-filter vacuums or damp-cleaning methods (wet mopping) are preferred over dry sweeping. Proper waste disposal and ventilation systems that prevent recirculation of contaminated air are also critical.
- Strengthening Host Health: A strong immune system improves resistance to PBFD. Stress minimization, nutritional support, and control of concurrent infections play a protective role. Birds with high HI antibody titers may resist disease development after exposure [20].

#### 9.6.1. Practical Guidelines for Breeders and Pet Owners

For Aviculturists

Aviculturists must adopt rigorous preventive measures to mitigate BFDV transmission within aviaries and breeding facilities. Essential guidelines include:

- Quarantine New Arrivals: Establish dedicated quarantine areas for incoming birds. New birds must be isolated for a minimum of 30 days and undergo BFDV testing (PCR on blood and feathers, supplemented by serological tests if available). Birds should only join the main collection after obtaining consecutive negative test results at both the start and end of quarantine [74,78].
- Protective Gear: When handling quarantined birds, caretakers must use disposable gloves, coveralls, and shoe covers. This gear should be replaced before entering resident bird areas. Ideally, separate personnel should handle isolated and resident populations to avoid fomite transmission.
- Routine Surveillance: Implement routine BFDV screening every 6–12 months, as asymptomatic carriers can persist undetected. Immediate testing is also essential when clinical signs arise, such as feather dystrophy or beak abnormalities [39].
- Dedicated Equipment: Maintain separate cleaning tools, feeding utensils, and housing items for each aviary group. Equipment must not be shared between enclosures unless thoroughly disinfected using proven virucidal solutions [78].
- Biosecure Facility Design: Limit facility access to essential staff. Require footbaths, handwashing, and protective clothing on entry. Use “all-in, all-out” bird movement protocols when possible. Visitors should be strictly limited or monitored, particularly around high-risk groups [39].

For Pet Owners

Pet bird owners can significantly reduce BFDV transmission risks at home and in the broader community by following these measures:

- Regular Disinfection: Weekly cleaning of cages, perches, bowls, and toys using bird-safe disinfectants like 1% Virkon S. Ensure complete drying before reuse.
- Hand Hygiene: Wash hands or use sanitizer before and after handling birds, especially when managing multiple individuals. This helps prevent fomite transfer of feather dust and viral particles [74,78].
- Avoid Sharing Items: Never share toys, bowls, or grooming tools between birds without thorough disinfection. Feather and fecal dust may contaminate porous materials.
- Observation and Prompt Action: Be alert to signs like feather loss, overgrown beak, or behavioral changes. If suspected, isolate the bird immediately and consult a veterinarian for PCR testing [77].
- Educate and Network: Stay informed about PBFD. Ensure pet shops, boarding services, and bird clubs enforce biosecurity. Encourage others to adopt safe practices [78].

#### 9.6.2. Prevention in Conservation and Restoration Programs

In conservation breeding or reintroduction programs involving endangered parrots, BFDV prevention is critical—particularly when the exclusion of infected individuals is unfeasible due to limited founder diversity. The following special considerations apply:

- Artificial Rearing Protocols: If breeding stock poses a BFDV risk, artificial incubation and hand-rearing of chicks under sterile conditions may be necessary to disrupt vertical or early postnatal transmission. This approach has been implemented successfully in species such as the Mauritius parakeet (*Psittacula eques*) and Hawaiian forest birds, especially where intensive disease management is required [74,85].
- Maximized Biosecurity: Facilities managing endangered parrots should enforce strict biosafety, including controlled human access, facility-wide disinfection protocols, and the physical separation of cohorts. Segregation of family lines in independent enclosures with separate airflow systems can mitigate intra-facility transmission risk [40].
- Pre-release Screening: All individuals destined for release must undergo comprehensive health assessments, including BFDV PCR testing. Releasing positive individuals into wild habitats may result in irreversible spillover events, as documented in the orange-bellied parrot (*Neophema chrysogaster*) where novel BFDV genotypes were introduced into critically endangered populations [13].
- Vaccination (Future Outlook): Although no commercial PBFD vaccine currently exists, experimental strategies using recombinant capsid proteins have shown promise in preliminary trials [7]. Conservation breeding programs may contribute to vaccine development by providing biological samples and participating in future field trials.

In summary, rigorous biosecurity, diligent monitoring, and emerging experimental vaccines represent the most effective PBFD management strategy for both captive and wild psittacine populations. These resource-intensive measures are vital for protecting endangered species against the additional threat posed by PBFD.

### 9.7. Vaccination Efforts

#### 9.7.1. Inactivated Virus Vaccines

Early experimental efforts toward vaccinating psittacine birds against beak and feather disease virus (BFDV) centered on the use of β-propiolactone-inactivated whole-virus preparations.

In one pivotal study, adult Umbrella cockatoos (*Cacatua alba*), Moluccan cockatoos (*C. moluccensis*), African grey parrots, and yellow-headed Amazon parrots (*Amazona oratrix*) were vaccinated intramuscularly or subcutaneously with β-propiolactone-inactivated BFDV. All adult birds seroconverted, developing hemagglutination inhibition (HI) and precipitating antibodies, demonstrating a strong immune response. Notably, chicks from vaccinated hens exhibited transient protection against BFDV challenge, remaining clinically healthy even when exposed shortly after hatching—suggesting maternal antibody-mediated immunity [86].

However, several limitations hinder the practicality of this approach:

- Viral Harvesting Requirements: Production of inactivated vaccines depends on harvesting BFDV from naturally infected birds, raising significant ethical concerns and biosafety risks.
- Carrier Risk and Shedding: While vaccinated birds may be protected from clinical disease, inactivated vaccines may not prevent viral replication or shedding. There is concern that vaccinated birds could still act as asymptomatic carriers [87].
- Scalability and Contamination: Vaccine production using field-derived viral isolates introduces the risk of contamination with other pathogens and presents challenges in scalability and standardization.

These early studies, while promising in terms of immunogenicity, emphasize the need for safer, scalable, and ethically acceptable vaccine strategies—leading to interest in recombinant protein and DNA vaccine approaches.

#### 9.7.2. Recombinant Subunit Vaccines

A promising modern approach to PBFD prevention involves recombinant expression of the BFDV capsid protein (Cap), which self-assembles into virus-like particles (VLPs) that mimic the native virus’s structure but lack infectious DNA.

Researchers have successfully produced BFDV Cap proteins using recombinant expression platforms such as baculovirus-infected insect cells and Nicotiana benthamiana plants. These recombinant Caps spontaneously self-assemble into VLPs resembling infectious virions, and they have been shown to retain functional hemagglutination and antigenicity, reacting with antibodies from naturally immune birds [22,65].

In a proof-of-concept study, N. benthamiana-expressed VLPs were produced using a bean yellow dwarf virus (BeYDV)-based vector system. Although protein yields were modest, VLPs formed correctly and resembled infectious particles under electron microscopy [65]. In animal trials, long-billed corellas (*Cacatua tenuirostris*) vaccinated with recombinant Cap protein (produced using a baculovirus system) developed specific anti-BFDV antibodies. Upon experimental challenge with live virus, vaccinated birds showed only transient viraemia and no feather lesions or persistent infection, in contrast to control birds who developed clinical disease [88].

Challenges remain with recombinant subunit vaccines. Achieving high-yield expression and consistent particle assembly remains a technical bottleneck. Some vaccine candidates have not fully prevented viral shedding or vertical transmission, requiring further optimization of adjuvants, formulations, and delivery routes. While plant systems offer cost-effective and scalable production, their expression levels for BFDV Cap remain lower than those in insect cells. Overall, recombinant subunit vaccines—particularly those based on VLPs—represent a scientifically validated and ethically viable path forward for PBFD prevention.

#### 9.7.3. Inactivated Adjuvanted Vaccines (Oil Emulsion)

Experimental oil-adjuvanted inactivated vaccines have been tested in species such as galahs (*Eolophus roseicapillus*), showing protection against PBFD.

In one study, adult and nestling galahs received primary single-oil and booster double-oil emulsion vaccines. Vaccinated nestlings showed robust antibody responses and were protected against acute PBFD after live virus challenge, whereas all unvaccinated controls developed clinical disease within four weeks [87,89].

However, challenges persist:

- Scaling up vaccine production remains resource intensive.
- Sourcing BFDV from infected birds raises biosafety and ethical concerns.
- Oil-adjuvanted formulations may cause injection-site reactions, particularly problematic in small-bodied species.

#### 9.7.4. DNA Vaccines

DNA vaccines encode the BFDV Cap protein and rely on the host’s cells to express antigen in situ. DNA vaccine constructs using mammalian expression vectors (e.g., pVAX1) have successfully induced specific antibody responses in psittacine birds in preclinical studies. Expression of Cap protein was confirmed in vitro, and immunogenicity was verified via ELISA and HI tests [90].

Limitations remain:

- Efficient gene delivery into avian cells is technically challenging.
- Avian-specific promoters and vector optimization are needed.
- Immunogenicity varies depending on expression levels and adjuvant use.

#### 9.7.5. Viral Vector Vaccines

Viral vectors (e.g., canarypox, adenovirus) are promising platforms for delivering BFDV antigens. Canarypox vectors have been used successfully in other avian diseases such as avian influenza and West Nile virus, providing proof-of-concept for safe gene delivery in birds [91]. To date, however, no published studies have documented successful canarypox- or adenovirus-based vaccines for PBFD. The strategy remains theoretical, pending further research on expression efficiency, host immunity, and safety in psittacines.

#### 9.7.6. Current Status and Challenges

As of this writing, no commercially licensed vaccine exists for PBFD. However, experimental studies have demonstrated the feasibility of vaccination, with recombinant subunit and DNA-based approaches showing partial protection against clinical disease and viral replication [88]. Despite this progress, several critical challenges remain:

- Efficacy: Although recombinant vaccines significantly reduce clinical signs and viral load, they do not achieve complete sterilizing immunity. Transient, low-level viral replication has been detected even in vaccinated birds [88]. This is likely due to BFDV’s capacity for immune evasion and persistent infection, a common feature of circoviruses.
- Duration of Immunity: Given that parrots can live for decades, vaccines must ideally confer long-term immunity. Most experimental vaccines, such as recombinant Cap subunits, show protection over a few months but lack long-term data on antibody durability or memory T-cell responses [90].
- Safety: No live-attenuated vaccines are currently under development due to concerns of reversion to virulence and interspecies transmission. Experimental DNA and subunit vaccines have shown good safety profiles in *psittacines*, including neonates [88].
- Genetic Diversity: BFDV displays significant global genetic variation, including recombinant strains, yet the Cap gene remains relatively conserved across genotypes, supporting its utility as a universal vaccine target [42].
- Production Scalability: While expression systems such as Pichia pastoris, baculovirus-insect cells, and Nicotiana benthamiana have been used to express BFDV Cap, protein yield remains a key bottleneck, especially in plant systems [22,65].

Ongoing research aims to:

- Improve antigen yield and particle assembly using plant or yeast expression systems.
- Evaluate novel adjuvants, including CpG oligonucleotides and nano emulsions, for better immune stimulation [90].
- Develop combinatorial immunization strategies (e.g., DNA prime–protein boost) to enhance immune breadth and longevity.

Despite current limitations, significant progress has been made. The foundation laid by experimental work in long-billed corellas, galahs, and other psittacine species underscores that a commercially viable vaccine is biologically feasible. Future breakthroughs will likely rely on integrating virology, immunology, and biotechnology to overcome existing hurdles.

## 10. Conservation and Policy Implications

PBFD poses not only a veterinary concern for pet and breeding parrots but also a growing conservation threat. All psittacine species are believed to be susceptible to BFDV, and more than one-third of parrot species are currently listed as threatened or endangered. As a result, PBFD can significantly disrupt conservation programs by infecting both captive breeding populations and wild individuals, particularly when undetected carriers are introduced through trade or reintroduction initiatives [9]. Spillover events into wild populations—including the critically endangered Orange-bellied Parrot (*Neophema chrysogaster*)—have been documented and underscore how disease introduction can imperil population recovery efforts [12,13].

As BFDV prevalence in wild populations increases, so too does the complexity of managing endangered species—especially in cases where habitat loss, hunting, or other stressors already threaten population viability [77]. Encouragingly, a growing body of literature has emerged to address PBFD through molecular surveillance, phylogenetic analysis, and improved diagnostic techniques [40]. To effectively mitigate PBFD’s impact, conservation strategies must integrate veterinary virology with wildlife management—particularly through stricter international trade regulations, expanded disease monitoring in wild populations, and improved biosecurity practices in captive breeding and reintroduction programs [39].

### 10.1. Global Spread and Surveillance in Wild Populations

As of 2016, PBFD had been confirmed in 33 countries. Since then, at least seven additional countries have reported PBFD cases, highlighting the virus’s expanding global presence.

- South Korea: The first officially confirmed PBFD case in South Korea was reported in a blue-and-yellow macaw in 2014 using PCR and sequencing-based diagnostics [45].
- United Arab Emirates (UAE): A 2016 study documented BFDV detection in the UAE in multiple psittacine birds using molecular testing methods [92].
- Chile: BFDV prevalence was confirmed in a 2019 study that detected infection in wild and captive parrots in Chile using PCR methods [33].
- Turkey: A 2020 study conducted in Turkey confirmed BFDV presence through sequence analysis of affected parrots [93].
- Mexico: The first peer-reviewed confirmation of PBFD in Mexico appeared in 2020, identifying BFDV in psittacines via PCR [94].
- Bangladesh: A 2022 study reported a 37% BFDV positivity rate among psittacine birds sampled from the pet trade, confirming active circulation of the virus in the region [32].
- Namibia: A 2023 molecular study confirmed BFDV infections in Namibian parrots, with sequencing results contributing to regional genetic data [95].

In regions where PBFD had previously been reported, ongoing surveillance continues to monitor infection dynamics and characterize circulating viral genotypes—especially within captive populations that may act as reservoirs for spillover into wild ecosystems [96]. These efforts are vital for understanding how BFDV evolves and spreads under natural and anthropogenic pressures.

Notably, novel genotypes of BFDV have emerged beyond traditional hotspots. In China, multiple genetically distinct BFDV lineages were isolated from budgerigars in commercial breeding facilities. Phylogenetic analysis revealed that these strains form unique clades, suggesting long-term undetected circulation or recent viral introductions followed by rapid divergence [69]. Similarly, early work in southern Africa reported genetically divergent strains that showed substantial differences from Australasian variants, pointing to regional genotype diversification [97].

Accordingly, key research priorities for global BFDV surveillance include:

- Sequencing newly identified strains and mapping their phylogenetic relationships to global lineages [44].
- Assessing PBFD prevalence in under-surveyed regions and species, especially those previously thought to be disease-free [98].
- Detecting genotypic shifts that suggest viral adaptation to novel hosts or environments [41].
- Advancing diagnostic technology, such as portable PCR systems, for rapid field deployment [99].

International phylogeographic tools and data-sharing platforms (e.g., GenBank) are increasingly being leveraged to trace global BFDV movement patterns and identify trade-associated transmission events [40]. These analyzes help clarify whether outbreaks in regions like Europe are genetically linked to strains circulating in Southeast Asia, thereby informing policy interventions along specific trade corridors.

### 10.2. Parrot Trade and Invasive Species: Spillover Dynamics

The international parrot trade boasts centuries-old origins; historically, parrots served as status symbols, and today, their intelligence and plumage maintain their global popularity as pets [100,101]. This trade, encompassing both legal and illegal activities, has resulted in parrots spreading far beyond their native habitats, with roughly one-quarter of traded parrots introduced into non-native regions [102,103]. Occasionally, these parrots escape or are intentionally released, forming established feral populations (e.g., urban parakeets globally) [104]. Approximately 10% of such introductions have led to invasive populations becoming self-sustaining. Notably, after Hurricane Andrew struck Florida (USA) in 1992, a large aviary’s destruction enabled numerous exotic parrots to escape, contributing significantly to local invasive populations.

Trade dynamics have evolved due to changing regulatory landscapes. As major markets such as the U.S. and Europe implemented import restrictions on wild parrots (e.g., the U.S. Wild Bird Conservation Act of 1992, the EU’s 2005 wild bird import ban), export activities shifted toward regions with looser regulations, notably parts of the Middle East and South Asia [102,105]. Concurrently, increased captive breeding in the U.S. and Europe met the demand for pet parrots domestically [106,107]. Although captive-bred parrots are generally less likely to establish invasive populations due to lower survival rates post-escape [108], they nonetheless represent significant potential vectors for disease [109].

Disease transmission remains a substantial concern within the global parrot trade. Conditions such as high-density housing during transportation, intermingling of diverse species in wholesale environments, and stress-induced immunosuppression collectively facilitate the spread of pathogens such as beak and feather disease virus (BFDV) [32,39]. Imported parrots—particularly those wild-caught or from intensive breeding operations—often act as asymptomatic carriers, introducing BFDV into new environments such as pet stores and aviaries, thereby exposing naïve bird populations [98]. In large-scale breeding facilities, a single infected bird can contaminate shared environments—such as nest boxes, food trays, and airspace—with viral particles [49]. This can lead to acute PBFD outbreaks in chicks, which are often removed without diagnosis, while infected adults may remain symptom-free yet continue to shed virus for extended periods [43,110]. Thus, captive breeding operations may inadvertently act as amplification hubs for BFDV, enabling continued transmission through commercial bird sales.

When BFDV-infected captive parrots escape or are deliberately released into the wild, they pose substantial risks to indigenous avian populations by facilitating viral transmission across species boundaries. In Brazil, escaped parrots carrying BFDV have the potential to infect endemic species such as Amazon parrots and macaws via shared foraging or roosting areas [32,39]. In Europe, invasive rose-ringed parakeets, originally from South Asia, have formed dense urban populations; if infected, their interactions with native birds at communal feeding stations or nesting cavities may serve as conduits for viral spillover [111]. Alarmingly, BFDV has also been detected in non-psittacine birds, such as finches and raptors, though it remains uncertain whether these species represent true viral reservoirs or incidental, non-transmitting hosts [98]. The introduction of BFDV through invasive parrots thus threatens not only native psittacine biodiversity but potentially broader avian communities. Clarifying the mechanisms and persistence of such cross-species transmission is critical for informed conservation policies, including potential removal or management of invasive parrot populations to mitigate ecological risk.

Policy-wise, regulating the global parrot trade remains complex. Although a complete international ban on parrot trade could hypothetically reduce the spread of infectious diseases such as PBFD, such a measure may unintentionally stimulate black market activity, thereby weakening formal health screening mechanisms [103]. While some countries require quarantine protocols and pathogen testing for imports, including PBFD screening, the enforcement of these policies is highly inconsistent across regions [40]. International conventions such as the Convention on Biological Diversity (CBD) call for the prevention of invasive species introductions, but implementation remains uneven due to conflicting national interests. For instance, some nations advocate stringent movement restrictions to reduce PBFD risk, while others prioritize economic gain through the pet trade and resist tighter controls. In this context, international collaboration is crucial. Improved information exchange on PBFD outbreaks, harmonization of quarantine standards (e.g., pre-export PBFD testing), and certification of PBFD-free breeding facilities could significantly enhance disease mitigation strategies [39]. Authorities such as CITES could integrate disease surveillance protocols directly into permitting systems, strengthening both conservation and biosecurity outcomes [96].

In conclusion, the global parrot trade, combined with resulting invasive populations, significantly contributes to the international dissemination of BFDV. Effective management strategies necessitate balancing rigorous trade regulations with enforcement to deter illicit activities and possibly implementing targeted invasive parrot population control measures to mitigate native species’ exposure risks.

### 10.3. PBFD-Related Policies and Management Programs

Recognizing the threat posed by PBFD, several governments have initiated specific policies or programs aimed at addressing the disease, particularly within the context of conserving endangered psittacine species.

Australia has been a global leader in developing policy responses to PBFD, reflecting both its status as a center of parrot biodiversity and the probable origin of beak and feather disease virus (BFDV) [39]. In 1999, PBFD was formally classified as a Key Threatening Process under the Environment Protection and Biodiversity Conservation (EPBC) Act, prompting the implementation of a Threat Abatement Plan (TAP) in 2002. The TAP introduced standardized diagnostic protocols—such as PCR-based BFDV detection—for conservation and management applications, promoted hygiene protocols in breeding centers, and called for long-term surveillance of wild psittacine populations [75]. However, the plan faced major operational hurdles, notably inadequate funding and weak enforcement capacity, which ultimately limited its effectiveness. By 2015, the TAP was deemed ineffective and replaced by non-binding threat abatement advice, a set of recommendations lacking legal authority or mandatory compliance. Australia’s trajectory underscores that even scientifically grounded and well-structured frameworks cannot yield sustained conservation outcomes without long-term political and financial commitment [103].

#### 10.3.1. Orange-Bellied Parrot (OBP) Recovery Program

The orange-bellied parrot (*Neophema chrysogaster*), one of Australia’s most critically endangered bird species, experienced a major setback in its captive breeding and reintroduction program during the early 2000s due to a widespread outbreak of PBFD. At the time, insufficient disease screening and lax biosecurity protocols permitted rapid transmission of BFDV within captive populations, resulting in significant mortality and a near-collapse of conservation efforts [39]. In response, the recovery program underwent substantial restructuring: breeding aviaries were relocated to virus-free facilities, routine molecular surveillance using PCR testing was institutionalized, and strict quarantine protocols were established for all incoming and outgoing bird transfers. These enhanced biosecurity measures not only curtailed further outbreaks but also contributed to improved reproductive outcomes and higher chick survival rates [96]. The OBP case highlights the critical importance of preemptive and sustained pathogen control within ex situ conservation programs and serves as a transferable framework for managing disease risk in other avian recovery efforts [112].

#### 10.3.2. Mauritius Parakeet Recovery Program

In Mauritius, conservation efforts to recover the critically endangered echo parakeet (*Psittacula eques*) have been significantly challenged by the persistent presence of PBFD. Following the detection of BFDV within the reintroduced population, management teams implemented biosecurity protocols such as disinfection of nest sites and targeted molecular screening of symptomatic individuals [39]. Despite these measures, disease control has been hindered by complex logistical issues, including inconsistent coordination among multiple stakeholder groups and the inherent difficulty of sustaining long-term epidemiological surveillance [96]. The co-occurrence of invasive rose-ringed parakeets (*Psittacula krameri*), which may act as viral reservoirs, further complicates risk management and raises concern over cross-species transmission [40]. The echo parakeet case exemplifies how even well-designed biosecurity strategies can be undermined by ecological complexity, host community interactions, and fragmented institutional governance structures.

In summary, although PBFD has received formal recognition within policy frameworks in select countries, notably Australia, the implementation and subsequent effectiveness of these measures have varied. Progression from isolated national efforts toward coordinated international strategies has been slow. Moving forward, future conservation initiatives should incorporate advanced diagnostic techniques, such as environmental DNA sampling for early detection of BFDV in wild populations, secure contingency funding for rapid response to outbreaks affecting critical populations, and clarify governance structures to facilitate stronger stakeholder collaboration. Only through such comprehensive and coordinated approaches can PBFD’s impacts on endangered parrot species be effectively mitigated.

## 11. Discussion and Conclusions

Psittacine beak and feather disease (PBFD) represents a significant veterinary and conservation challenge with a global distribution. Its causative agent is the beak and feather disease virus (BFDV), a circovirus affecting multiple psittacine species and some non-psittacines. Despite decades of research, key knowledge gaps persist. These gaps include mapping the full host range; elucidating molecular mechanisms of latency in asymptomatic carriers; and clarifying how viral evolution facilitates immune escape. Such knowledge gaps directly hinder the development of viable therapeutics and have so far prevented the formulation of an effective, commercially available vaccine.

The international trade in parrots, both legal and illicit, is a primary driver of BFDV’s cross-continental spread. This global circulation has created a ‘melting pot’ of novel BFDV genotypes. Consequently, even countries like Australia, where the virus is considered endemic, face an elevated threat from the reintroduction of exotic viral strains to which local populations may lack immunity. The risk is amplified by significant regional disparities in policy and enforcement, particularly in major trade hubs. For instance, while some African breeding operations report quantifiable economic impacts (e.g., annual flock losses of 10–20%), and Asia’s role in the parrot trade expands with minimal BFDV-specific regulations, coordinated screening policies remain absent. The vulnerability of high-stakes conservation programs to such systemic risks is starkly illustrated by the challenges facing Australia’s critically endangered orange-bellied parrot, which remains at risk despite significant domestic management efforts.

Reliable diagnosis remains the linchpin of any control program. Effective BFDV control is further impeded by critical diagnostic and therapeutic bottlenecks. Viral latency, where carriers may test negative intermittently using standard PCR protocols, complicates disease screening and population management. This diagnostic uncertainty is exacerbated by national quarantine frameworks that are often poultry-centric and lack mandatory BFDV testing for imported psittacines. Therefore, advancing reliable diagnostic protocols and effective prophylactic tools is essential to break transmission cycles. While no commercial vaccine is yet available, research into recombinant protein and DNA vaccine platforms is progressing, with recent studies demonstrating preliminary efficacy in vivo.

This review synthesizes current knowledge on PBFD for a broad audience, including veterinarians, conservation biologists, government policymakers, and aviculturists. It underscores that resolving the dual challenges of viral latency in diagnostics and defining the complete host spectrum are immediate research priorities. Ultimately, translating this scientific understanding into actionable, integrated global strategies through sustained collaboration among these diverse stakeholders is imperative for the future conservation of psittacine species.

## Figures and Tables

**Table 1:** Clinical signs of PBFD.

Stage	Clinical Signs
Newly hatched chicks	Diarrhea, lethargy, crop stasis, anorexia; no feather signs observed; death within 1–2 weeks
Fledgling birds	Similar signs to newly hatched chicks; abnormal feathers observed; death within 1–2 weeks
Chronic cases (6 months to several years)	Feathers become twisted, split, and fall out; beak abnormalities; death within 6 months to 2 years

**Table 2:** Various diagnostic methods for PBFD with pros and cons.

Methods	Description	Pros	Cons
HA	Hemagglutination assay (HA) is a method used to measure the ability of viruses or antibodies to cause red blood cells (erythrocytes) to clump or agglutinate. It is frequently employed to quantify viral particles or evaluate antigen–antibody interactions within diagnostic and research settings [14,59,63].	Simplicity, Low cost, Quick Result	Specificity, Sensitivity, Variable Results
HI	Hemagglutination inhibition (HI) assay measures the presence of antibodies against viruses in serum by observing if they prevent the clumping of red blood cells [14,59,63].	Specific Detection, Simplicity, Reliable	Limited Spectrum, Interference, Time Consuming
Histology	Histology in pathology involves the microscopic examination of tissue samples to diagnose disease. This analysis is critical for determining the nature, severity, and potential treatment of various medical conditions [56].	Special Stains	Time consuming, Technique Sensitivity, Sampling Bias
Standard PCR	PCR (Polymerase Chain Reaction) is a technique used to amplify specific DNA sequences, enabling the detection and quantification of DNA samples [14,59,63].	Specificity, Sensitivity, Established	Contamination, Size Limitation
WGS	Whole-genome sequencing (WGS) is a method that determines the complete DNA sequence of an organism’s genome at a single time [71].	Comprehensive, High Resolution, Outbreak Tracking	High Cost, Data Overload

## Data Availability

The original data presented in the study are openly available in ICTV at https://doi.org/10.1007/s00705-022-05516-5 (accessed on 30 June 2025).
